# Vitamin D Regulates Cytokine Patterns Secreted by Dendritic Cells to Promote Differentiation of IL-22-Producing T Cells

**DOI:** 10.1371/journal.pone.0130395

**Published:** 2015-06-24

**Authors:** Andrea Sommer, Mario Fabri

**Affiliations:** 1 Department of Dermatology, University of Cologne, Cologne, Germany; 2 Center for Molecular Medicine Cologne, University of Cologne, Cologne, Germany; University Medical Center of the Johannes Gutenberg University of Mainz, GERMANY

## Abstract

One central mechanism, by which vitamin D regulates human immune responses, is the direct modulation of dendritic cells (DCs). However, the effect of vitamin D on several key DC functions, such as the secretion of central inflammatory cytokines, remains controversial. Moreover, whether vitamin D treatment of DCs regulates their ability to promote differentiation of IL-17-/IL-22-producing T cell subsets, such as Th17 and Th22 cell, is not known. Here, we report that vitamin D treatment during differentiation of monocytes into DCs markedly enhanced their ability to secrete TNF-α, IL-6, IL-1β and IL-23. Cytokines secreted by vitamin D-treated DC were significantly more potent in driving differentiation of IL-22-producing T cells, but not IL-17-producing T cells, as compared to secreted cytokines of not-vitamin D-treated DCs. Finally, we found that the differentiation of IL-22-producing T cells mediated by supernatants of vitamin D-treated DCs was dependent on TNF-α IL-6 and IL-23. In summary, our study suggests a novel role of vitamin D in regulating DC-mediated immune responses in humans.

## Introduction

Dendritic cells (DCs) are pivotal to the instruction of immune responses in humans. In this regard, immunogenic DCs secrete TNF-α, IL-6 and IL-1β, pro-inflammatory cytokines, which have a wide spectrum of biological activities that help to coordinate the immune response, for instance in the context of infection [1]. TNF-α, IL-6 and IL-1β recruit other immune cells to the site of inflammation and, together with IL-12 and IL-23, regulate the polarization of effector T cells, in particular into Th1, Th17 and Th22 cells. While Th1 cell-derived IFN-γ is central in triggering host defense pathways against intracellular pathogens, it has become increasingly clear that Th17 and Th22 cells, at least in part by secreting IL-17 and IL-22, promote host defense responses against extracellular pathogens for instance by upregulating antimicrobial peptides at epithelial surfaces [2–4]. However, in contrast to IFN-γ, IL-22 is not considered an exclusively inflammatory cytokine, given its important role in promoting wound healing and ensuring tissue homeostasis [5–7]. In addition, IL-17 production is not only restricted to pro-inflammatory T cells given that regulatory T cells can express IL-10 in conjunction with IL-17 [8, 9]. Of note, other cell types, for example lymphoid tissue inducer (LTi) cells, γδ T cells and natural killer (NK) cells produce IL-17a and IL-22 [10]. However, the functional importance of this production during infection and homeostasis is not well understood [11].

Development of Th1 cells is induced by IL-12p70, which consists of the IL-12p40 and the IL-12p35 subunits [12–15]. The requirements for human Th17 development are not completely resolved, however, critical roles for IL-6, IL-1β, and IL-23 (consisting of the IL-12p40 and the IL-23p19 subunits), as well as of TGF-β have been documented [16–22]. Moreover, it was shown that TNF-α, IL-6 and IL-23 promoted human IL-22-producing T cell differentiation [23–28]. Whether TGF-β inhibits differentiation of IL-22-producing T cells remains controversial [23, 29, 30]. In contrast to immunogenic DCs, tolerogenic DCs are characterized by secretion of higher levels of anti-inflammatory cytokines, in particular IL-10, and they promote the development of anti-inflammatory Th2 and regulatory T cell (Treg) responses to limit inflammation [31].

In the last years, it has become increasingly clear that vitamin D hormone is a major regulator of human immune responses [32]. Pre-vitamin D is produced in the skin from 7-dihydroxycholesterol upon UVB irradiation [33]. After UVB irradiation vitamin D is synthesized over several days even in the absence of additional UV light [34, 35]. Vitamin D is hydroxylated to 25D-hydroxy-vitamin D (25D), mainly in the liver, by cytochrome P450 enzymes, including the CYP27a1- and CYP2r1-hydroxylase [36]. Subsequently, 25D is hydroxylated by the 1-α-hydroxylase CYP27b1, mainly in the kidney, to generate 1,25D. However, other human tissues locally produce relevant amounts of bioactive 1,25D. For instance, skin keratinocytes expressing CYP27b1 and DCs expressing CYP27a1 and CYP27b1 are able to locally produce 1,25D [37–43]. Moreover, CYP27b1 is activated in human macrophages by innate and acquired immune stimuli and efficiently catalyzes the conversion of 25D to 1,25D [44, 45].

A major mechanism by which vitamin D modulates immune responses is the regulation of DC functions [46]. It is believed that vitamin D treatment of human DCs elicits an anti-inflammatory/tolerogenic Th2/Treg-promoting, but not an inflammatory/immunogenic Th1-promoting DC phenotype [47–55]. This has been linked to the finding that vitamin D-treated DCs secrete a higher ratio of IL-10/IL-12. Moreover, several studies found that vitamin D-treated DCs showed upregulated expression of immune-modulatory surface molecules, for instance ILT3 (CD85k) or PD-L1 (CD274), both B7 family members that negatively regulate T cell activation, as well as downregulated expression of stimulatory molecules, such as HLA-DR and CD80 [47–55]. Together, the immune alterations in vitamin D-treated DCs *in vitro* provide one mechanistic explanation for the observation that clinically vitamin D deficiency is associated with an increased risk for autoimmune diseases [56]. However, vitamin D deficiency has also been associated with an increased risk for infections [56], in which pro-inflammatory cytokines and Th1, but also Th17 and Th22 cell responses are important for protective immune defense. The effect of vitamin D on the secretion of central host defense cytokines by DCs, including TNF-α, IL-6, IL-1β, IL-23, as well as the role of vitamin D in regulating DC-mediated differentiation of IL-17- and IL-22-producing T cells remain unclear. Here, we addressed the question whether vitamin D treatment of human DCs influences their ability to regulate inflammatory and protective host defense responses.

## Materials and Methods

### Reagents

Recombinant human granulocyte-macrophage colony-stimulating factor (rGM-CSF), interleukin-4 (rIL-4), and interleukin-2 (rIL-2) were purchased from Miltenyi. The Toll-like receptor ligand 2/1 (TLR2/1L) Pam_3_Cys-SKKK was from EMC Microcollections, the TLR4L LPS (Escherichia coli 0111:B4) from Sigma-Aldrich and human soluble CD40 ligand trimer (CD40L) from Peprotech. 1,25D and 25D were obtained from Biomol and used at a final concentration of 10^−8^ M and 10^−7^ M, respectively. For staining of surface molecules the following monoclonal antibodies were purchased: FITC-labeled anti-HLA-DR (clone G46-6, BD Bioscience), PE-labeled anti-CD80 (clone HII49, BD Bioscience), APC-labeled anti-CD1a (clone L307.4, BD Bioscience), PerCP-labeled anti-CD14 (clone TÜK4, Miltenyi), FITC-labeled anti-CD206 (clone DCN228, Miltenyi), PE-labeled anti-CCR5 (clone NP-6G4, ebioscience), PE-labeled anti-CCR7 (clone FR11-11E8, Miltenyi), PE-labeled anti-ILT3 (clone REA141, Miltenyi), APC-labeled anti-PD-L1 (clone MIH1, ebioscience); as corresponding isotypes the following monoclonal antibodies were used: FITC-labeled IgG2a (clone G155-178, BD), FITC/PE/PerCP/APC-labeled IgG1 (clone MOPC-21, BD). PMA and Ionomycin were obtained from Sigma-Aldrich and Brefeldin A from BD Bioscience. For intracellular cytokine staining the following monoclonal antibodies were used: PE-labeled anti-IL-22 (clone 22URTI, eBioscience), PerCP-Cy5.5-labeled anti-IFN-γ (clone B27, BD Biosciences), Alexa Fluor 647-labeled anti-IL-17a (clone N49-653, BD Biosciences), APC-labeled anti-IL-4 (clone 8D4-8, BD Biosciences). The following monoclonal blocking antibodies were purchased from R&D Systems: anti-TNF-α (clone 6401), anti-IL-6 receptor-α (clone 17506), anti-TGF-β (clone 9016), anti-IL-12/IL-23p40 (clone 24901), anti-IL-1β (clone 2805). Fetal calf serum (FCS) and human AB serum were purchased from PAA. Baseline 25D levels in FCS were 1,5x10^-8^ M. Physiologic levels of 1,25D in FCS are very low. All vitamin D reagents were tested to be endotoxin free using Limulus amebocyte lysate testing (Lonza).

### Ethics statement

This study was conducted according to the principles expressed in the Declaration of Helsinki. The study was approved by the local Ethic Committee (Ethikkommission) of the University of Cologne, Germany. All donors provided written informed consent for the collection of peripheral blood and subsequent analysis.

### Cell isolation and DC culture

Whole blood or ‘buffy coats’ from healthy donors were obtained with informed consent. PBMCs were isolated by Ficoll-Paque (GE Healthcare). Monocytes were isolated via MACS cell separation (Miltenyi) according to the manufactures instructions either using the monocyte untouched isolation kit II for DC surface molecule analyses or using the CD14^+^ microbeads for PCR experiments and to obtain supernatants for the T cell differentiation assays. DCs were differentiated by culturing 0.5x10^6^/ml monocytes in rGM-CSF (200 U/ml) and rIL-4 (100 U/ml) in RPMI with 10% FCS. The serum was either supplemented with 1,25D (10^−8^ M) or 25D (10^−7^ M) or used without additional 1,25D or 25D. At day six or seven DCs were either stained for the expression of antigen-presenting and co-stimulatory surface molecules or were stimulated with Pam_3_Cys-SKKK (1 μg/ml), LPS (10 ng/ml), or CD40L (5 μg/ml) in fresh media containing 10% FCS supplemented with 1,25D (10^−8^ M) or 25D (10^−7^ M) or used without addition of 1,25D or 25D. After 18–24 hours, supernatants were harvested from these cultures and the DCs were analyzed for surface molecule expression by FACS using a Calibur (BD Biosciences) and FlowJo software (Tree Star). We did not observe different DC numbers after differentiation in FCS supplemented with 1,25D (10^−8^ M) or 25D (10^−7^ M), as compared to differentiation in FCS alone. Supernatants were immediately frozen for future culture with T cells. TNF-α, IL-6, IL-1β, IL-12p40, IL-12p70, IL-23 and IL-10, as well as total and active TGF-β levels in the supernatants were determined by ELISA or CBA (BD Biosciences). To measure the total amount of TGF-β in DC supernatants samples were pre-treated with 1 M HCL for 5 minutes followed by neutralization with 1.2 M NaOH.

### T cell differentiation assay

T cell differentiation assays were carried out as previously described [23] with slight modifications: Naïve CD4^+^ T cells were isolated from whole blood or ‘buffy coats’ from healthy donors by naïve CD4^+^ T cell isolation kit (Miltenyi) according to the manufacturer’s instruction. 5x10^4^ T cells were stimulated using 1:1 anti-CD3/anti-CD28 antibody-coated beads according to the manufacturer’s instruction in the T cell expansion and activation kit (Miltenyi) in 96-well U-bottom plates and cultured in 10% human AB serum, 50% 1,25D^diff^-DC or serum-DC supernatant, and 40% fresh media. In blocking experiments anti-TNF-α, anti-IL-6 receptor-α, anti-TGF-β, anti-IL-1β and anti-IL-12/IL-23p40 monoclonal antibodies alone or in combination were added to the cultures at the indicated concentrations. On day five, rIL-2 was added at a final concentration of 50 U/ml and T cells were cultured for additional seven days. For intracellular cytokine staining T cells were re-stimulated with PMA (50 ng/ml) and Ionomycin (0.5 μg/ml) for five hours, for the final 2.5 hours in the presence of Brefeldin A, in fresh media. In some experiments T cells were counted using Trypan blue exclusion and were stained for intracellular cytokines using the Cytofix/Cytoperm fixation/permeabilization solution kit (BD Biosciences) according to the manufacturer’s instruction. For measuring secreted cytokines T cells were re-stimulated with PMA (50 ng/ml) and Ionomycin (0.5 μg/ml) for 18–24 hours (without addition of Brefeldin A) in fresh media. Cytokines in the T cell culture supernatants were measured by ELISA or CBA (BD Biosciences). To measure cytokine expression of naïve T cells after isolation (day 0) 5x10^4^ naïve CD4 T cells were stimulated with PMA (50 ng/ml) and Ionomycin (0.5 μg/ml) or cultured in media alone immediately after isolation, for five hours, the final 2.5 hours in the presence of Brefeldin A, and subsequently stained for intracellular cytokines using the Cytofix/Cytoperm fixation/permeabilization solution kit (BD Biosciences).

### PCR

mRNA was isolated from the GM-CSF/IL-4 stimulated monocytes after 24h using the RNeasy mini kit (Qiagen) according to the manufacturer’s recommended protocol. cDNA was prepared and mRNA levels assessed by qPCR as previously described [45]. Primer sequences for human cathelicidin, CYP24A1 and h36B4 were previously reported [44, 45].

### Statistics

*P*-values were calculated using two-tailed Student’s *t*-tests. All treatment groups in each subfigure were conducted in parallel. *n* refers to the number of repeated experiments performed with cells from individual human donors.

## Results

### Vitamin D treatment during DC differentiation promotes secretion of pro-inflammatory cytokines

**Fig 1:**
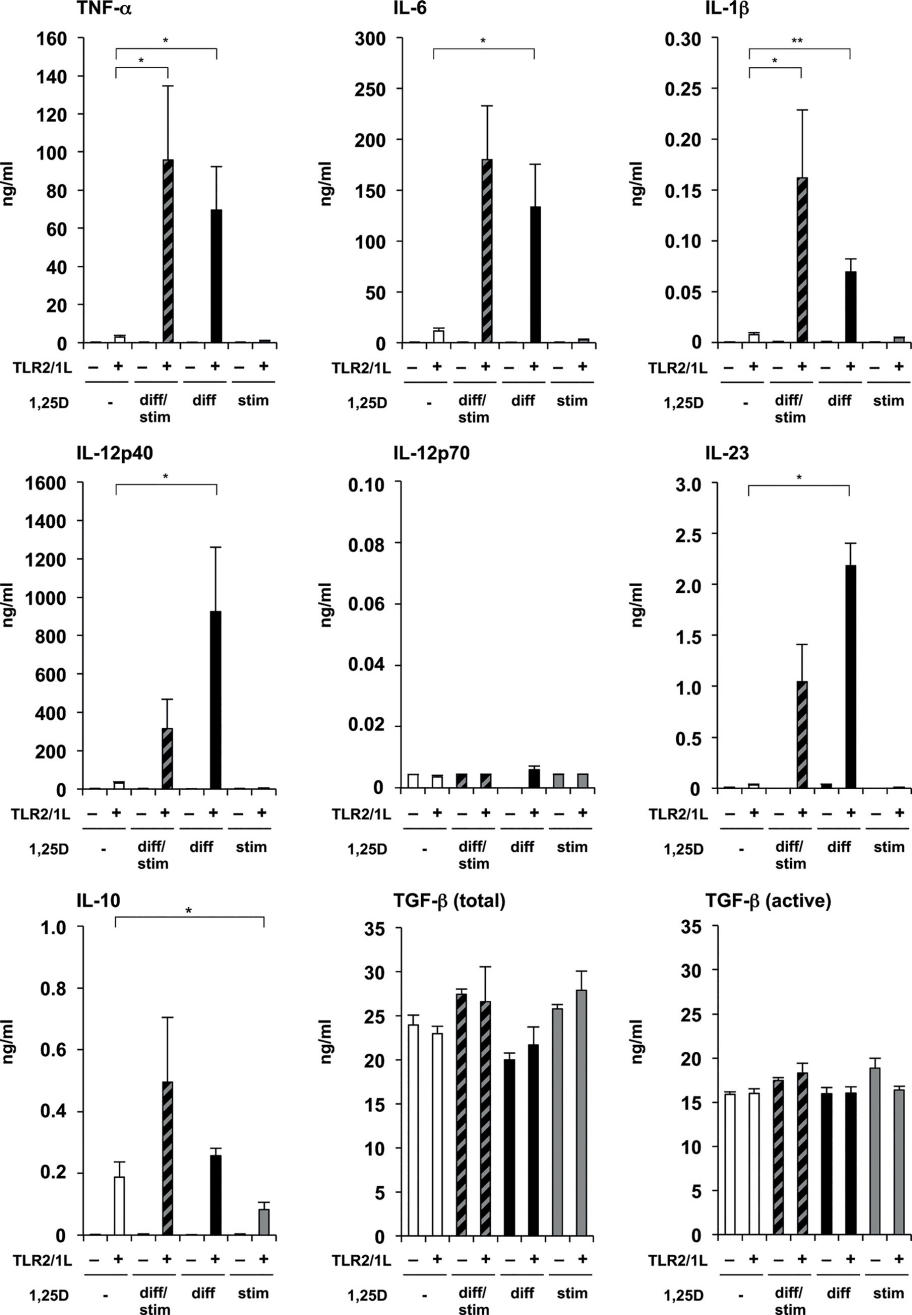
Effect of 1,25D on differentiation and/or stimulation of monocyte-derived DCs. Monocytes were differentiated for six days with rGM-CSF and rIL-4 in media with 10% FCS in the presence (1,25D^diff^-DCs or 1,25D^diff/stim^-DCs) or absence (serum-DCs or 1,25D^stim^-DCs) of additional 1,25D (10^−8^ M). Subsequently, 1,25D-DCs and serum-DCs were stimulated with TLR2/1L (1 μg/ml) or left untreated in the presence (1,25D^diff/stim^-DCs or 1,25D^stim^-DCs) or absence (serum-DCs or 1,25D^diff^-DCs) of additional 1,25D (10^−8^ M) and cultured in fresh media with 10% FCS for 18–24 hours. TNF-α, IL-6, IL-1β, IL-12p40, IL-12p70, IL-23 and IL-10, as well as total and active TGF-β levels in culture supernatants were measured by ELISA or CBA (mean cytokine levels in ng/ml ± SEM, n = 4). Results shown in Fig. 1 and Fig 2 were conducted using cells from the same cell preparations of identical donors. *p<0.05, **p<0.01

Given the pivotal role of TNF-α, IL-6, IL-1β, IL-12 and IL-23, as well as TGF-β and IL-10 in regulating inflammation and T cell differentiation, we investigated the effect of vitamin D on the secretion of these cytokines by DCs. Specifically, we analyzed the effect of vitamin D on the differentiation, as well as the stimulation of human DCs. Therefore, primary human monocytes were *in vitro* differentiated into DCs in 10% FCS in the presence (1,25D^diff^-DCs) or absence of additional 1,25D (10^−8^ M). Alternatively, *in vitro* differentiated DCs (without additional vitamin D during differentiation) were stimulated by the TLR2/1 ligand (TLR2/1L) Pam_3_Cys in the presence (1,25D^stim^-DCs) or absence of additional 1,25D (10^−8^ M). Furthermore, we analyzed the overall effect, when additional 1,25D (10^−8^ M) was present during the differentiation and stimulation of DCs (1,25D^diff/stim^-DCs). 1,25D^diff^-DCs as compared to DCs not treated with additional vitamin D (serum-DCs), produced significant higher amounts of TNF-α (70 ng/ml vs. 3 ng/ml, p<0.05), IL-6 (133 ng/ml vs. 12 ng/ml, p<0.05), and IL-1β (69 pg/ml vs. 8 pg/ml, p<0.01)(Fig 1) in response to TLR2/1L. They also secreted more IL-12p40 (924 ng/ml vs. 28 ng/ml, p<0.05) and IL-23 (2184 pg/ml vs. 31 pg/ml, p<0.05)(Fig 1). Moreover, we did not measure any significant difference in IL-10 secretion, or total or active TGF-β (Fig 1). In contrast, 1,25D^stim^-DCs secreted significantly lower amounts of IL-1β (5 pg/ml vs. 8 pg/ml, p<0.05) and IL-10 (82 pg/ml vs. 187 pg/ml, p<0.05)(Fig 1) than serum-DCs in response to TLR2/1L, and showed a trend towards lower TNF-α, IL-6, IL-12p40, and IL-23 secretion (Fig 1). IL-12p70 secretion was low and not significantly different (Fig 1). Moreover, we did not measure any difference in total or active TGF-β (Fig 1). The cytokine pattern of 1,25D^diff/stim^-DCs was similar to the cytokine pattern observed in 1,25D^diff^-DC cultures (Fig 1). To show VDR activation in our model system we tested for induction of key vitamin D genomic targets, cathelicidin and CYP24A1, and found that both genes were significantly induced on mRNA level by 1,25D treatment (10^−8^ M)(Figure A and B in S1 Fig).

**Fig 2:**
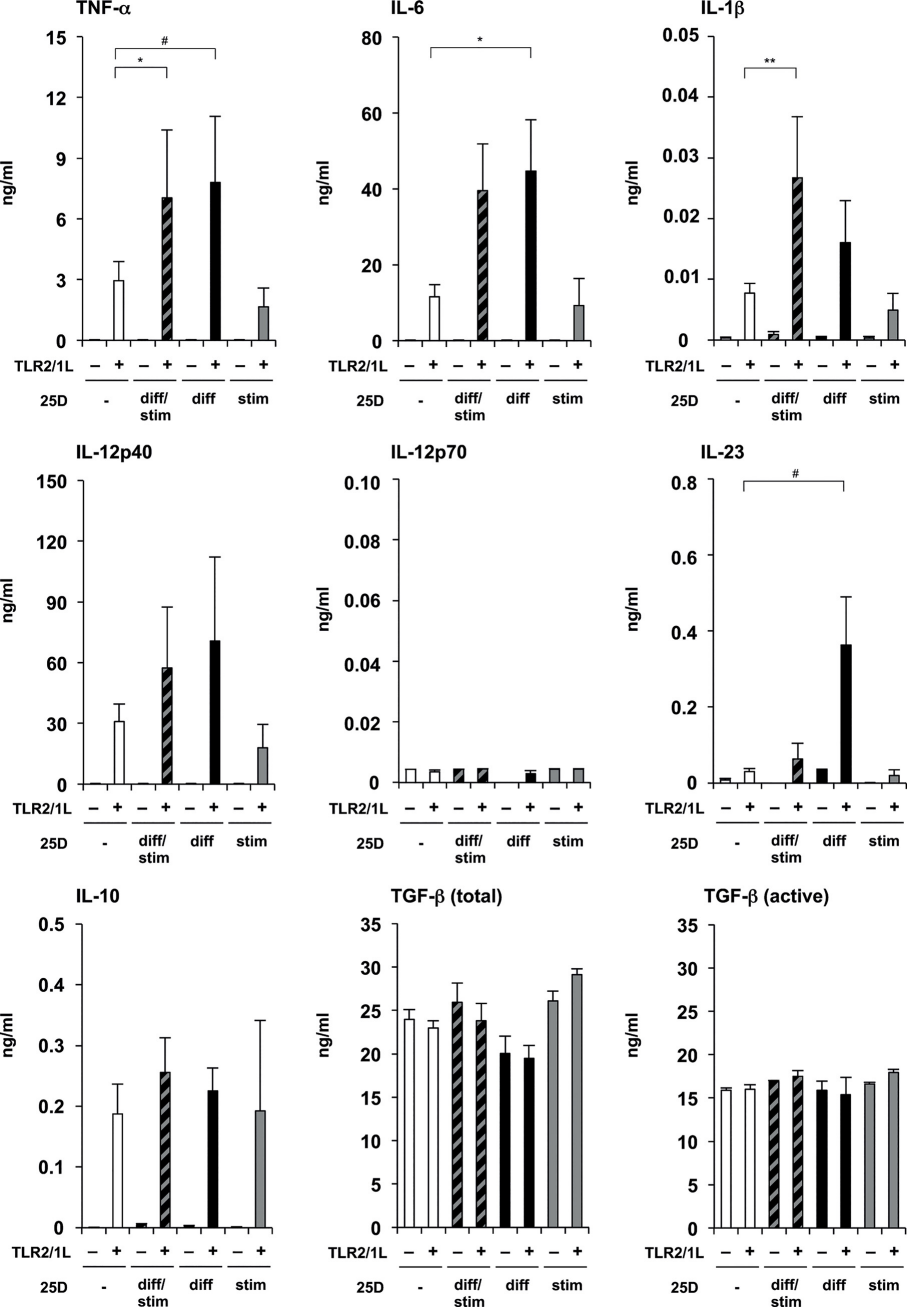
Effect of 25D on differentiation and/or stimulation of monocyte-derived DCs. Monocytes were differentiated for six days using rGM-CSF and rIL-4 in media with 10% FCS in the presence (25D^diff^-DCs or 25D^diff/stim^-DCs) or absence (serum-DCs or 25D^stim^-DCs) of additional 25D (10^−7^ M). Subsequently, 25D-DCs and serum-DCs were stimulated with TLR2/1L (1 μg/ml) or left untreated in the presence (25D^diff/stim^-DCs or 25D^stim^-DCs) or absence (serum-DCs or 25D^diff^-DCs) of additional 25D (10^−7^ M), and cultured in fresh media with 10% FCS for 18–24 hours. TNF-α, IL-6, IL-1β, IL-12p40, IL-12p70, IL-23 and IL-10, as well as total and active TGF-β levels in culture supernatants were measured by ELISA or CBA (mean cytokine levels in ng/ml ± SEM, n = 4). Results shown in Fig 1 and Fig. 2 were conducted using cells from the same cell preparations of identical donors. *p<0.05, **p<0.01, ^#^p≤0.05

Given that 1,25D levels are low and kept relatively constant in human serum, most immune cells in peripheral tissues rely on the local conversion of 25D into bioactive 1,25D [32]. Thus, we also investigated the effect of additional 25D (10^−7^ M)(Fig 2) on the differentiation and/or stimulation of human DCs in the same experimental setup as for 1,25D. We detected more TNF-α 8 ng/ml vs. 3 ng/ml, p<0.05), IL-6 (45 ng/ml vs. 12 ng/ml, p<0.05) and IL-23 (364 pg/ml vs. 31 pg/ml, p<0.01)(Fig 2) secretion, as well as a trend towards more IL-1β and IL-12p40 secretion upon stimulation via TLR2/1 by 25D^diff^-DCs as compared to serum-DCs (Fig 2). In contrast, we did not measure any significant difference in IL-10 secretion, or total or active TGF-β (Fig 2). With respect to 25D^stim^-DCs there was no significant difference in cytokine secretion as compared to serum-DCs. Moreover, the cytokine pattern secreted by 25D^diff/stim^-DCs was comparable to the pattern secreted by 25D^diff^-DCs.

**Fig 3:**
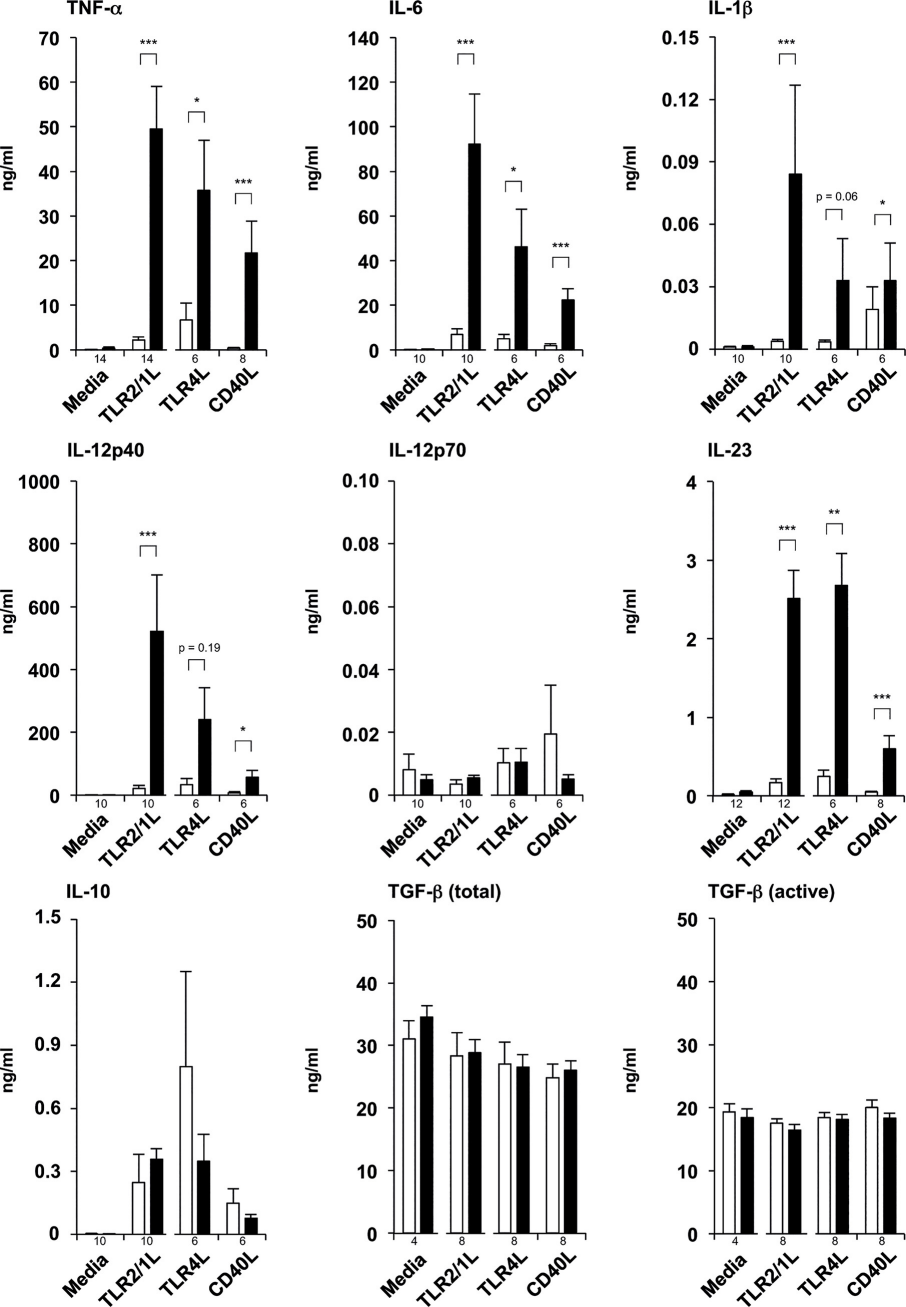
Cytokine profiles of 1,25D^diff^-DCs vs. serum-DCs stimulated with different ligands. Monocytes were differentiated for six days using rGM-CSF and rIL-4 in media with 10% FCS in the presence (1,25D^diff^-DCs, black bars) or absence (serum-DCs, white bars) of additional 1,25D (10^−8^ M). Subsequently, 1,25D^diff^-DCs and serum-DCs were stimulated with TLR2/1L (1μg/ml), TLR4L (10 ng/ml) or CD40L (5 μg/ml) or left untreated, and cultured in fresh media with 10% FCS for 18–24 hours. TNF-α, IL-6, IL-1β, IL-12p40, IL-12p70, IL-23 and IL-10, as well as total and active TGF-β levels in culture supernatants were measured by ELISA or CBA (mean cytokine levels in ng/ml ± SEM, n of each condition indicated by numbers under the bars). *p<0.05, **p<0.01, ***p<0.001

Next, we asked if differences between the cytokine patterns observed in 1,25D^diff^ DCs vs. serum-DCs are dependent on the activating stimulus. Thus, we compared cytokine secretion of 1,25D^diff^-DCs vs. serum-DCs upon activation with the TLR2/1L Pam_3_Cys, the TLR4L LPS, or CD40L. We found that all three ligands induced significantly more TNF-α and IL-6 secretion by 1,25D^diff^-DCs as compared to serum-DCs (Fig 3). 1,25D^diff^-DCs also secreted more IL-1β, IL-12p40 and IL-23 irrespective of the activating stimulus (Fig 3). IL-12p70 secretion was low and not different between 1,25D^diff^-DCs and serum-DCs (Fig 3). Moreover, we did not measure significantly different amounts of IL-10, or total or active TGF-β (Fig 3). Taken together, our data show that vitamin D treatment of differentiating DCs increases their secretion of TNF-α, IL-6, IL-1β, IL-12p40 and IL-23.

To characterize the vitamin D-treated DCs in more detail, we analyzed the profile of surface molecule expression on vitamin D-treated DCs in comparison to serum-DCs before and after additional stimulation by TLR2/1L. On un-stimulated 1,25D^diff^-DCs we detected a significant lower expression of the antigen-presenting molecules HLA-DR and CD1a, as well as the co-stimulatory molecule CD80 as compared to serum-DCs (S2 & S3 Figs). The same pattern was observed for 25D^diff^-DCs compared to serum-DCs, yet only reaching statistical significance for CD1a (S2 & S3 Figs). In addition, while 1,25D^diff^-DCs and 25D^diff^-DCs showed a trend towards lower expression of the mannose-receptor CD206 (S2 & S3 Figs), CD14 was upregulated as compared to serum-DCs (S2 & S3 Figs). Moreover, the chemokine receptors CCR5 (CD195) and CCR7 (CD197), involved in trafficking of DCs either to inflamed tissue or to second lymphoid organs, were unaffected by 1,25D (10^−8^ M) and 25D (10^−7^ M) treatment during the DC differentiation process (S2 & S4 Figs). In contrast, 1,25D^diff^-DCs and 25D^diff^-DCs expressed significantly higher levels of ILT3 and PD-L1 (S2 & S4 Figs). TLR2/1L treatment resulted in stimulation of all DC types (1,25D^diff^-DC, 25D^diff^-DC, serum-DC) as measured by up-regulation of HLA-DR and CD80 when comparing TLR2/1-stimulated to the un-stimulated individual DC types (S2 & S3 Figs). Nevertheless, the expression pattern of HLA-DR, CD80, CD1a and CD14 as well as ILT3 in 1,25D^diff^-DCs and 25D^diff^-DCs as compared to serum-DCs within the TLR2/1-stimulated DC group was very similar to those in un-stimulated DCs. Also the mannose receptor CD206 and the chemokine receptors CCR5 and CCR7 remained at a low expression level within the stimulated DC group comparable to the un-stimulated DC group. Of note, PD-L1 was induced by TLR2/1L stimulation in all DC types (S2 & S4 Figs). In summary, vitamin D treatment of differentiating DCs resulted in a dual pro-/anti-inflammatory phenotype characterized by a tolerogenic expression pattern of cell surface molecules, yet a markedly enhanced secretion of pro-inflammatory cytokines.

### Supernatants of 1,25D^diff^-DCs promote differentiation of IL-22-producing T cells

**Fig 4:**
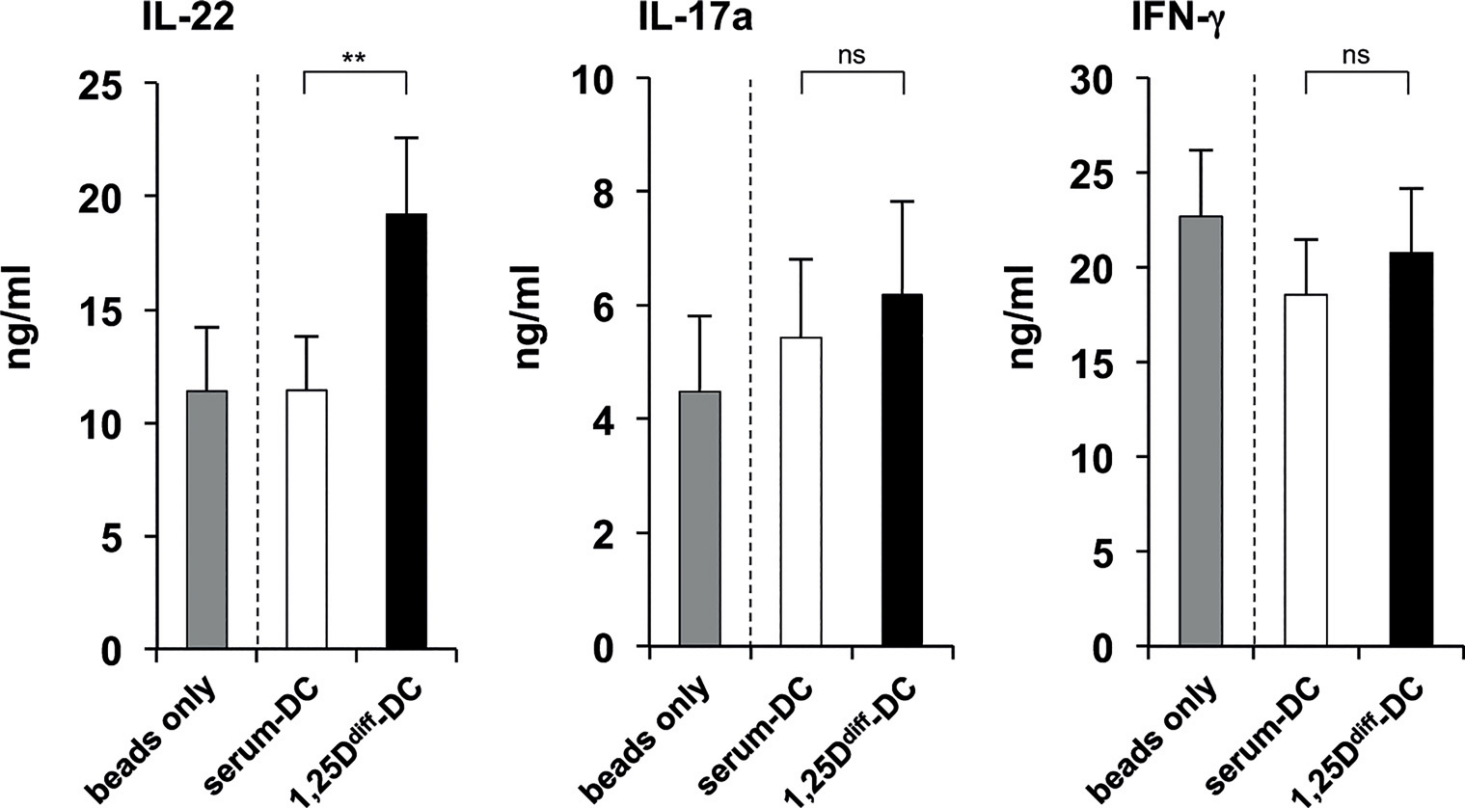
Supernatants of 1,25D^diff^-DCs promote differentiation of IL-22-secreting T cells. Monocytes were differentiated into DCs with rGM-CSF and rIL-4 in media with 10% FCS in the presence (1,25D^diff^-DCs) or absence (serum-DCs) of additional 1,25D (10^−8^ M). 1,25D^diff^-DCs and serum-DCs were stimulated with TLR2/1L (1 μg/ml) in fresh media and supernatants were collected after 18–24 hours. Subsequently, TLR2/1-induced 1,25D^diff^-DC and serum-DC supernatants were added to naïve CD4^+^ T cells activated with CD3/CD28-coated beads. As a control, naïve CD4^+^ T cells were incubated with CD3/CD28-coated beads without addition of DC supernatants (beads only). After five days, rIL2 was added to all cultures. On day 12, T cells were re-stimulated with PMA/Ionomycin in fresh media and cytokine secretion evaluated after 18–24 hours. Levels of T cell-derived IL-22, IL-17a and IFN-γ were assessed by ELISA (mean of cytokine levels in ng/ml ± SEM, IL-22 n = 15, IL-17a/IFN-γ n = 13). *p<0.05, **p<0.01

One of the key functions of pro-inflammatory cytokines is the instruction of T cell polarization into effector T cells. Because it was shown that TNF-α and IL-6 promoted development of IL-22-producing T cells, and a combination of TNF-α, IL-6 and IL-1β promoted development of IL-17-producing T cells [23], we asked whether vitamin D treatment of differentiating DCs would enhance their ability to induce IL-17- and/or IL-22-producing T cells. Therefore, we activated 1,25D^diff^-DCs and serum-DCs with TLR2/1L and collected the supernatants of these cultures at 18–24 hours. Subsequently, we added the 1,25D^diff^-DC and serum-DC supernatants to naïve CD4^+^ T cells activated via their TCR. After culture for 12 days to allow differentiation into different T cell subsets, T cells were re-stimulated in fresh culture media for 18–24 hours and T cell cytokine secretion into the culture medium analyzed by ELISA or CBA. We found that T cells differentiated in the presence of TLR2/1-induced 1,25D^diff^-DC supernatants secreted significantly more IL-22 as compared to T cells cultured in the presence of TLR2/1-induced serum-DC supernatants (19.2 ng/ml vs. 11.4 ng/ml, p<0.01)(Fig 4). In contrast, we detected no significant differences in secretion of IFN-γ and IL-17a (Fig 4), as well as IL-4 (Fig. A in S5 Fig), a signature cytokine for Th2 cell phenotypes, but a trend towards less IL-10, a signature cytokine for Tregs, respectively (Fig. D in S5 Fig). However, in the presence of 1,25D^diff^-DC supernatants differentiated T cells secreted significantly more TNF-α as their serum-DC supernatant-treated counterparts (36.9 ng/ml vs. 19.9 ng/ml, p<0.05)(Fig. E in S5 Fig).

**Fig 5:**
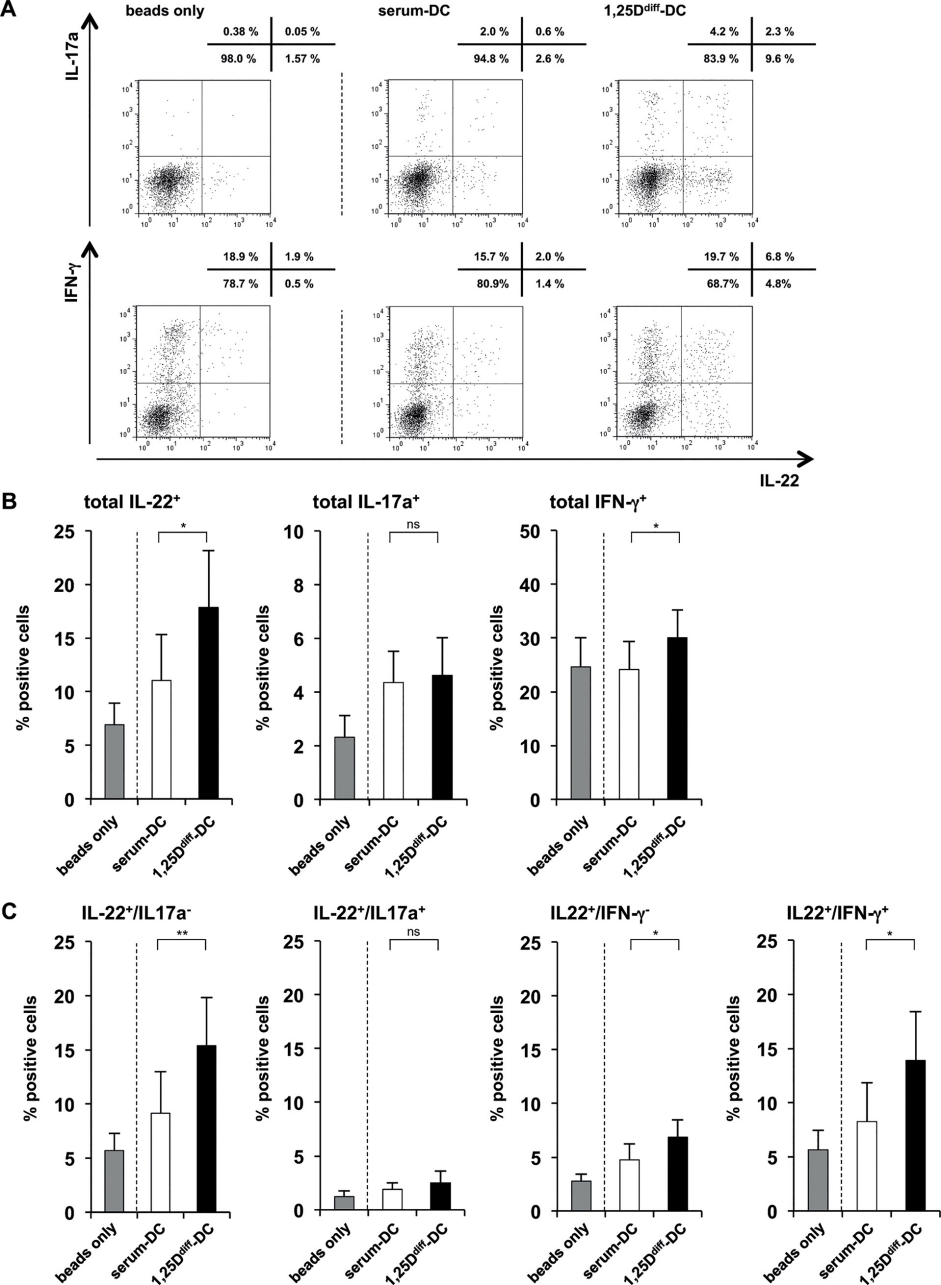
Supernatants of 1,25D^diff^-DCs promote differentiation of IL-22-expressing T cells. Supernatants of TLR2/1-induced 1,25D^diff^-DCs and serum-DC were added to naïve CD4^+^ T cells activated with CD3/CD28-coated beads (as described in Fig 4). After five days, rIL2 was added to all cultures. On day 12, T cells were re-stimulated with PMA/Ionomycin for five hours, the last 2.5 hours of culture in the presence of Brefeldin A, in fresh media and intracellular cytokine expression of IL-22, IFN-γ or IL-17a was measured. **(A)** Dot plots from one representative staining of one donor out of eleven. Upper panel of dot plots shows co-expression of IL-17a and IL-22, lower panel shows co-expression of IFN-γ and IL-22. Numbers above each dot plot indicate frequency of positive cells in each quadrant. **(B)** Frequency of total IL-22-, IL-17a- and IFN-γ-expressing CD4^+^ T cells assessed by intracellular cytokine staining (mean percentage of positive cells ± SEM, n = 11). **(C)** Frequency of IL-22^+^/IL-17a^+^ and IL-22^+^/IL-17a^-^ or IL-22^+^/IFN-γ^+^ and IL-22^+^/IFN-γ^-^ CD4^+^ T cells assessed by intracellular cytokine staining (mean percentage of positive cells ± SEM, n = 11). *p<0.05, **p<0.01

Next, we investigated the effect of 1,25D-treatment (10^−8^ M) on the expression of the T cell signature cytokines, IFN-γ, IL-17a, IL-22 and IL-4, by intracellular cytokine staining. Therefore, after culture for 12 days with supernatants of TLR2/1-activated 1,25D^diff^-DCs and serum-DCs, T cells were re-stimulated in fresh culture media for 5 h, 2.5 h in the presence of Brefeldin A, and T cell phenotypes evaluated by FACS. Culture of naïve CD4^+^ T cells with 1,25D^diff^-DC supernatants resulted in significantly more IL-22^+^ T cells as compared to T cells cultured in supernatants of serum-DCs (17.9% vs. 11.0% IL-22^+^ T cells, p<0.05)(Fig 5A & 5B). In contrast, we observed no significant differences in the frequency of IL-17a^+^ and IL-4^+^ T cells, and a modest, yet significant increase in IFN-γ^+^ T cells in these cultures (30.0% vs. 24.2% IFN-γ^+^ T cells, p<0.05)(Fig 5A and 5B & Fig. B in S5 Fig). Of note, in the starting naïve T cell population (day 0) IL-22- and IL-17a-producing T cells were almost absent (0.11% IL-22^+^ and 0.08% IL-17^+^ T cells) and frequencies of IFN-γ^+^ T cells were low (3.36% IFN-γ^+^ T cells)(Figs. A and B in S6 Fig). Because at least three human Th subsets produce IL-22: T cells co-expressing IFN-γ and IL-22 (Th1/Th22 cells), T cells co-expressing IL-17a and IL-22 (Th17 cells), both referred to as “non-classical” Th22, and T cells that produce IL-22, but neither IFN-γ nor IL-17a (*bona fide* Th22 cells) [7, 23, 29, 57–59], we also performed double stainings for IL-22/IFN-γ and IL-22/IL-17a. When T cells were cultured in 1,25D^diff^-DC supernatants, more T cells showed IL-22^+^/IFN-γ^-^ (6.9% vs. 4.8% IL-22^+^/IFN-γ^-^ T cells, p<0.05) and IL-22^+^/IL-17a^-^ phenotypes (15.4% vs. 9.1% IL-22^+^/IL-17a^-^ T cells, p<0.01) as compared to T cells cultured in serum-DC supernatants (Fig 5A & 5C). Moreover, we observed significant higher percentages of IL-22^+^/IFN-γ^+^ T cells (13.9% vs. 8.3% IL-22^+^/IFN-γ^+^ T cells, p<0.05), when cells were cultured in 1,25D^diff^-DC vs. serum-DC supernatant (Fig 5A & 5C). However, the frequency of IL-22^+^/IL-17a^+^ was not different in the two groups (Fig 5A & 5C). Besides, no significant difference was observed in the frequency of IL-22^+^/IL-4^+^ T cells (Fig. C in S5 Fig). Of note, the number of T cells was significantly higher in all conditions as compared to the initiation of the culture (day 0, S7 Fig) demonstrating proliferation of T cells. In summary, our data show that cytokines secreted by activated 1,25D^diff^-DCs promote the differentiation of IL-22-producing, but not IL-17-producing T cells.

### 1,25D^diff^-DC-supernatant mediated differentiation of IL-22-producing T cells is dependent on TNF-α/IL-6 and IL-23

**Fig 6:**
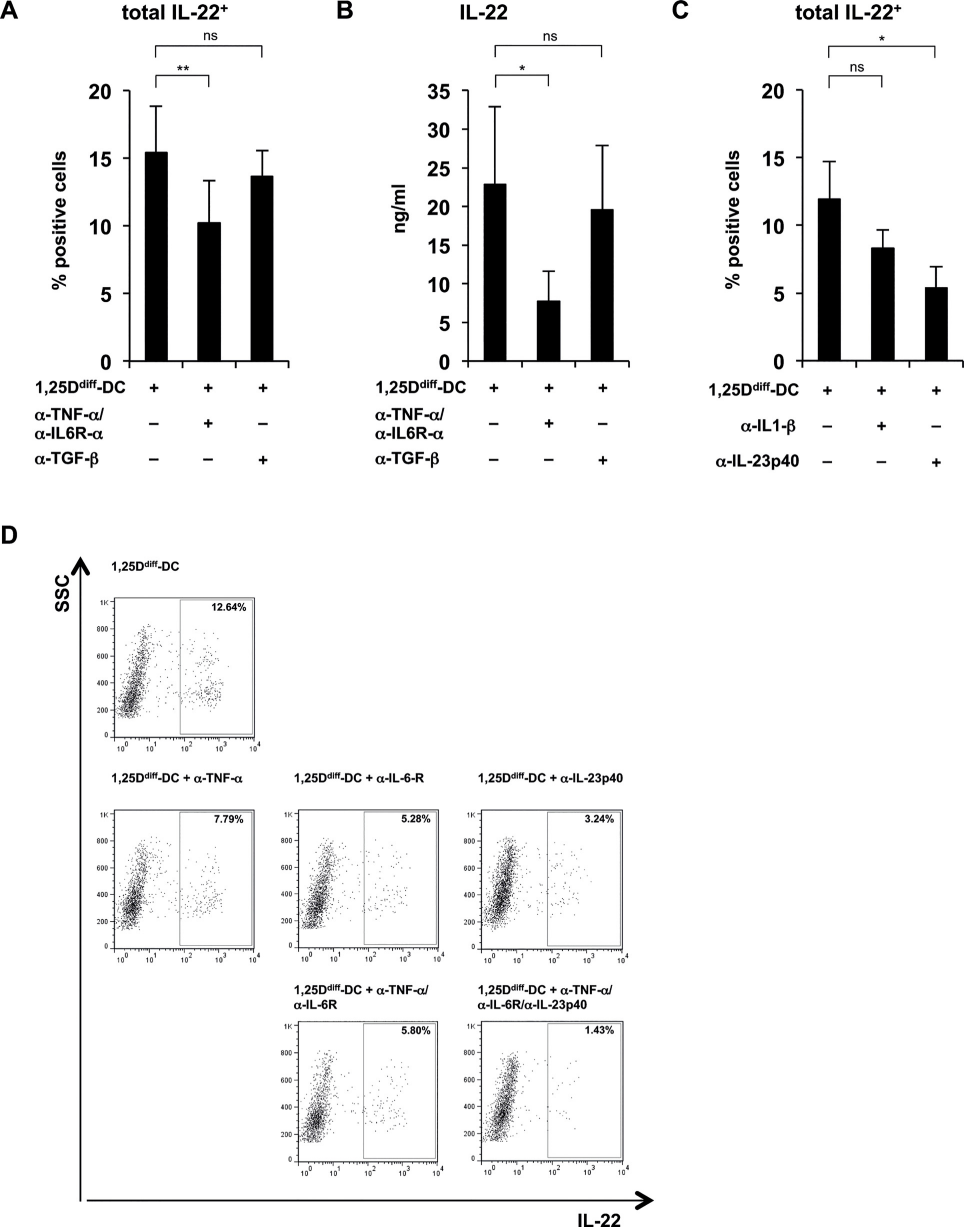
1,25D^diff^-DC-supernatant mediated priming of IL-22-producing T cells is dependent on TNF-α IL-6 and IL-23. Supernatants of TLR2/1-stimulated 1,25D^diff^-DCs were added to naïve CD4^+^ T cells activated via CD3/CD28-coated beads (as described in Fig 4) in the presence or absence of different monoclonal blocking antibodies as indicated. After five days, rIL2 was added to all cultures. On day 12, T cells were restimulated with PMA/Ionomycin for five hours, the last 2.5 hours of culture in the presence of Brefeldin A, in fresh media and intracellular cytokine expression of IL-22, IFN-γ or IL-17a was measured. Cytokine secretion was evaluated after 18–24 hours without further addition of Brefeldin A. **(A)** Anti-TNF-α, anti-IL-6R-α (5 μg/ml each) or anti-TGF-β (10 μg/ml). T cell-derived IL-22 assessed by ELISA (mean of cytokine levels in ng/ml ± SEM, n = 5). **(B)** Anti-TNF-α, anti-IL-6R-α (5 μg/ml each) or anti-TGF-β (10 μg/ml). Frequency of total IL-22-expressing CD4^+^ T cells assessed by intracellular cytokine staining (mean percentage of positive cells ± SEM, n = 5). **(C)** Anti-IL-1β or anti-IL-23p40 (5 μg/ml each). Frequency of total IL-22-expressing CD4^+^ T cells assessed by intracellular cytokine staining (mean percentage of positive cells ± SEM, n = 5). **(D)** Anti-TNF-α, anti-IL-6R-α or anti-IL-23p40 (5 μg/ml each) blocking antibodies alone or in combination. Dot plots from one representative staining of one donor out of five showing the frequency of IL-22-expressing CD4^+^ T cells against the sideward-scatter (SSC). Numbers in rectangle gate indicate frequency of positive cells. *p<0.05, **p<0.01

TNF-α and IL-6 have been shown to be sufficient, as well required in the DC-mediated induction to drive development of IL-22-producing T cells [23]. Thus, we hypothesized that priming of IL-22-producing T cells by 1,25D^diff^-DCs is dependent on TNF-α/IL-6. To test our hypothesis we cultured activated, naïve CD4^+^ T cells with supernatants from TLR2/1-stimulated 1,25D^diff^-DCs in the presence of monoclonal anti-TNF-α/anti-IL-6R-α neutralizing antibodies. As a control we used a monoclonal anti-TGF-β neutralizing antibody. After culture for 12 days, we performed intracellular cytokine staining for IL-22. Blocking TNF-α/IL-6R-α, but not TGF-β, significantly inhibited the frequency of IL-22^+^ T cells (15.4% vs. 10.2% IL-22^+^ T cells, p<0.01)(Fig 6A). We also investigated cytokine secretion by the T cells and found that the addition of anti-TNF-α/anti-IL-6R-α antibodies resulted in a significant reduction of IL-22 secretion (22.9 ng/ml vs. 7.8 ng/ml, p<0.05)(Fig 6B). In contrast, blocking TGF-β did not have any significant effect (Fig 6B). Next, we explored the influence of IL-23, reported to play an important role in IL-22^+^ T cell commitment [24–28], as well as IL-1β. Blocking of IL-23, via the IL-12p40 subunit, significantly inhibited the frequency of IL-22^+^ T cells (11.9% vs. 5.4% IL-22^+^ T cells, p<0.05)(Fig 6C). However, blocking IL-1β did not have any significant effect (Fig 6C). Taken together, our data showed that 1,25D^diff^-DC-secreted TNF-α, IL-6 and IL-23 contributed to the development of IL-22 expressing T cells. To further characterize the individual and combined effects of TNF-α, IL-6 and IL-23 in this process, we performed additional blocking experiments. Blocking of IL-6 alone was more efficient than blocking TNF-α alone (12.6% vs 5.3% vs. 7.8%)(Fig 6D) and as potent as the combined blocking of TNF-α and IL-6 (12.6% vs 5.8%)(Fig 6D). In addition, blocking of IL-23 via the IL-12p40 subunit in combination with TNF-α/IL-6R-α was more potent than the individual effects and resulted in drastic inhibition of the differentiation of IL-22^+^ T cells (12.6% vs. 1.4% IL-22^+^ T cells)(Fig 6D). Of relevance, given the lack of a monoclonal blocking antibody specific for the IL-23p19 subunit, we cannot exclude that the inhibition of IL-12p70 signaling, due to blocking of the IL-12p40 subunit, has also an effect on inhibition of IL-22^+^ T cell differentiation in our setup. In summary, these data show that differentiation of IL-22-producing T cells by supernatants of 1,25D^diff^-DCs is dependent on TNF-α IL-6 and IL-23.

## Discussion

In this study, we report that vitamin D treatment during differentiation of human DCs increased their ability to promote aspects of host protective immunity. A key finding was the strong enhancing effect of vitamin D on TNF-α, IL-6, IL-1β, as well as IL-23 secretion by DCs. In contrast, we did not detect major differences in IL-12p70, IL-10 or TGF-β secretion. Together, these data show that vitamin D treatment of differentiating human DCs favors a pro-inflammatory cytokine profile, a fact that, even though reported for TNF-α, IL-6, IL-23 in the past [47, 48, 53, 54], has not been interpreted as such [60]. Strikingly, the cytokines secreted by TLR2/1L-induced 1,25D^diff^-DCs were potent in driving differentiation of IL-22^+^ CD4^+^ T cells, demonstrating a novel role of vitamin D in regulating DC-mediated instruction of T cell responses. In addition, we demonstrate that the 1,25D^diff^-DC-mediated instruction of IL-22^+^ CD4^+^ T cells was dependent on TNF-α/IL-6 and IL-23. Our findings stand in accordance with several current reports using different models, including mouse and human. TNF-α and IL-6 alone or in combination induced IL-22 production from naïve T cells [23, 24]. Moreover, collective evidence indicates that IL-23 contributes essentially to the induction of IL-22 production from different immune cells, including CD4^+^ T cells [24–27, 61–64]. However, the exact role of different cytokine combinations in driving differentiation of IL-22^+^ T cell remains controversial [59], but it seems likely that this depends on the experimental setup and/or biological context. In our experiments, TGF-β, although secreted, seemed not to significantly regulate differentiation of IL-22-producing T cells [23, 29, 59], because neutralizing TGF-β in 1,25D^diff^-DC supernatants had no effect. Of note, we targeted the IL-12-/IL-23-shared p40 subunit as an IL-23p19 specific monoclonal blocking antibody was not available. Therefore, we are not able to rule out that IL-12p70 could also contribute to IL-22^+^ T cell differentiation. Of relevance, even though vitamin D treatment of differentiating DCs enhanced their secretion of IL-1β [65] and IL-23, both of which have been linked to the development and maintenance of Th17 cells [16–18], in our *in vitro* T cell differentiation model, we did not measure an increase in Th17 cells. However, it is noteworthy that the relative and absolute amounts of *DC-secreted* IL-1β and IL-23 detected in our cultures were 25 to 100 times lower than the amounts of *recombinant* cytokines used in previous protocols [16–18], which may explain the apparent differences.

Given the lack of an established pathogen-DC-T cell co-culture system to investigate differentiation of IL-22-producing cells from *naïve* T cells, we adopted a model, in which T cell are activated by CD3/CD28-coated beads and cultured with DC supernatants according to Duhen *et al*. [23], a key paper showing that TNF-α and IL-6 drive differentiation of IL-22-producing T cells. Therefore, our study does not experimentally take contact-dependent mechanisms into account, yet addressed differences in T cell instruction by vitamin D-treated vs. serum-DCs in a bystander fashion. Interestingly, even though DCs drove IL-22^+^ T cell differentiation in a classical mixed-lymphocyte reaction and in an APC-CD3-autologous T cell co-culture, direct contact of DCs and T cells seemed not to be required for this process [23, 59]. In fact, cytokines were sufficient to drive IL-22^+^ T cell commitment in CD3/CD28-activated T cells [7, 23, 29, 59], consistent with our presented data. Nevertheless, it will be interesting in future studies to investigate the effect of vitamin D on critical steps in DC antigen-presentation to naïve T cells using extracellular antigens, including specific antigen-binding and-uptake, as well as intracellular processing (involving degradation by proteasomes and lysosomal-associated proteases, loading on MHC molecules, etc.) and quality of MHC:peptide complexes, co-stimulation etc. [66, 67].

During immune responses in human tissues, such as the skin, monocytes are recruited to the site of inflammation, where they locally differentiate into effector macrophages and DCs [68, 69]. Thus, one could speculate that the vitamin D effect on differentiating DCs provides a mechanism, by which vitamin D amplifies the initial inflammatory immune responses in the context of infection. In contrast, when we stimulated already differentiated DCs in the presence of vitamin D, we observed a tendency towards decreased secretion of inflammatory cytokines consistent with previous reports [49, 52]. Thus, one could speculate that under steady-state conditions, inflammatory responses by tissue DCs are suppressed by vitamin D, preventing exaggerated inflammation and tissue destruction. Moreover, we observed a dual pro-inflammatory/anti-inflammatory phenotype of vitamin D-treated DCs characterized by a tolerogenic expression pattern of cell surface molecules consistent with previous studies [47, 49, 51, 53–55, 70, 71], but also by a markedly enhanced secretion of pro-inflammatory cytokines. This could suggest that monocytes, within a continuous spectrum of DC-macrophage polarization, differentiate more towards a macrophage phenotype in the presence of vitamin D [72]. Besides, the dual inflammatory/anti-inflammatory phenotype implies that vitamin D promotes the initiation of an innate inflammatory response and at the same time could balance the acquired immune response. In this regard, an increased production of T cell IL-22 may result in restoration of tissue homeostasis and in combination with TNF-α in induction of antimicrobial peptides [3, 5, 73, 74]. Generally, IL-22 promotes epithelial innate immune mechanisms, which can either be harmful or protective: IL-22 contributes to host defense against extracellular bacterial infections, tissue homeostasis and inflammation, in particular at epithelial barriers like bowel, lung and skin [75]. In detail, IL-22 has been described to have pro- and anti-inflammatory activities depending on the context, e.g. the specific tissue microenvironment, the infectious agent and the cytokine milieu, in which IL-22 is expressed [76]. For example, while IL-22 can act synergistically with IL-17a in promoting pathological airway inflammation [76], both contribute to protective antimicrobial peptide expression in the skin [63, 77]. Also, the co-secretion of TNF-α and IL-22 was essential to trigger antimicrobial peptide expression in keratinocytes, and was shown to be the optimal combination for the skin immune response against *Candida albicans* in a 3D-skin model [3, 7]. Therefore, especially the induction of ‘polyfunctional’ Th22 cells, T cells co-expressing IL-22 in conjunction with other cytokines, seems to be critical. We found that 1,25D^diff^-DCs promoted differentiation of total IL-22^+^ T cells, IL-22/IFN-γ co-expressing T cells and an enhanced TNF-α secretion by T cells. Furthermore, we did not observe an increase in IL-4 expression and secretion, or IL-10 secretion. Of interest, compatible with two previous human studies, IL-4 was only co-expressed by a small fraction of IL-22^+^ T cells [58, 59]. However, our findings on IL-4 and IL-10 stand in contrast to a mouse study, showing an increased IL-4 and IL-10 expression by murine CD4^+^CD45RB^high^ naïve T cells, isolated from spleens of OVA_323-339_-specific TCR-transgenic DO11.10 mice and stimulated with OVA-peptide loaded mitomycin-treated wild-type splenocytes, when 1,25D was added to the co-culture [58, 59, 78]. This probably reflects species differences, or differences in the experimental setup. Nevertheless, our data suggest that human vitamin D-treated DCs, by enhancing differentiation of IL-22^+^ and IL-22^+^/IFN-γ^+^ T cells, as well as TNF-α secretion by T cells, can contribute to host defense responses at epithelial surfaces [3].

The effect of vitamin D on DCs could cooperate with other vitamin D-mediated mechanisms that promote protective host defense responses. We and others have previously shown that vitamin D was required for the human host defense response against intracellular pathogens [44, 45, 79, 80]. Moreover, vitamin D induced expression of the skin-homing receptor CCR10 on human T cells [23, 40].

Without any doubt further investigations including animal models are needed to decipher the role of vitamin D in regulating IL-22 T cell responses *in vivo*. Nevertheless, our findings are relevant for the clinical use of vitamin D-treated GMP-produced DCs [81, 82]. On one hand, if used as therapeutics in autoimmune diseases the effect of vitamin D on the pro-inflammatory cytokine production by DCs could aggravate aspects of inflammation. On the other hand, increased production of IL-22 and TNF-α could not only promote tissue homeostasis in inflammatory diseases [30, 83, 84], but also regulate host defense by inducing antimicrobial peptide production at epithelial barriers in infections [3, 5, 6].

Clinically, vitamin D deficiency has paradoxically not only been linked to poorer outcomes in autoimmunity, but also in infectious diseases [56]. Nevertheless, the fact that many immune mechanisms, which contribute to detrimental inflammation in autoimmunity are identical to those that mediate host protection, has raised questions, if and how, vitamin D can promote protective acquired immune responses in the context of infections. One explanation could be derived from the concept that the reported promoting effect of vitamin D on the innate macrophage response in the context of infection [44, 45, 79, 80] simply outweighs potential inhibitory effects on the acquired response, and/or that vitamin D provides a negative feed-back loop on acquired immunity to limit excessive inflammation. However, in the present study, we provide evidence that human DCs differentiated in the presence of vitamin D do not solely exhibit an anti-inflammatory phenotype. In fact, they are superior to serum-DCs in secreting key host defense cytokines and promoting differentiation of IL-22-producing T cells in a bystander manner, thereby indicating that vitamin D promotes aspects of both pro-inflammatory and anti-inflammatory immune responses in humans.

## Supporting Information

S1 Fig 1,25D induces key genomic targets in differentiating DCs. Primary human monocytes were cultured for 24h with rGM-CSF and rIL-4 in media with 10% FCS in the presence (1,25D^diff^-DC) or absence of 1,25D (10^−8^ M) (serum-DC). **(A)** Cathelicidin, **(B)** CYP24A1 gene expression was assessed by qPCR (arbitrary unit (AU) ± SEM, n = 7–8). **p<0.01
(EPS)

S2 Fig Expression of cell surface molecules on 25D^diff^-DCs and 1,25D^diff^-DC and serum-DCs. Monocytes were isolated and differentiated into DCs with rGM-CSF and rIL-4 in media with 10% FCS in the presence (25D^diff^-DCs, 1,25D^diff^-DCs) or absence (serum-DCs) of 25D (10^−7^ M) or 1,25D (10^−8^ M), respectively. DCs were stained on day 6 before or on day 7 after TLR2/1L (1 μg/ml) stimulation for 24h. Surface molecule expression was evaluated by FACS. Expression levels of HLA-DR, CD1a, CD80, CD14, CD206, CCR5, CCR7, ILT3 and PD-L1 on 25D^diff^-DCs and 1,25D^diff^-DC and serum-DCs (Δmean fluorescence intensity (ΔMFI = MFI^specific monoclonal antibody^—MFI^corresponding isotype control^) ± SEM, n = 7). *p<0.05, **p<0.01, ***p<0.001
(EPS)

S3 Fig Surface expression of HLA-DR, CD1a, CD80, CD14 and CD206 on 25D^diff^-DCs and 1,25D^diff^-DC and serum-DCs. Monocytes were isolated and differentiated into DCs with rGM-CSF and rIL-4 in media with 10% FCS in the presence (25D^diff^-DCs, 1,25D^diff^-DCs) or absence (serum-DCs) of 25D (10^−7^ M) or 1,25D (10^−8^ M). DCs were stained on day 6 before or on day 7 after TLR2/1L (1 μg/ml) stimulation for 24h. Expression of surface molecules was evaluated by FACS. Histograms from one representative staining of one donor out of seven (grey shaded area: specific antibody, black solid line: isotype control).
(EPS)

S4 Fig Surface expression of CCR5, CCR7, ILT3 and PD-L1 by 25D^diff^-DCs and 1,25D^diff^-DC and serum-DCs. Monocytes were isolated and differentiated into DCs with rGM-CSF and rIL-4 in the presence (25D^diff^-DCs, 1,25D^diff^-DCs) or absence (serum-DCs) of 25D (10^−7^ M) or 1,25D (10^−8^ M). DCs were stained on day 6 before or on day 7 after TLR2/1L (1 μg/ml) stimulation for 24h. Surface molecule expression was evaluated by FACS. Histograms from one representative staining of one donor out of seven (grey shaded area: specific antibody, black solid line: isotype control).
(EPS)

S5 Fig Effect of 1,25D^diff^-DC supernatant on T cell-derived IL-4, IL-10 and TNF-α. Activated naïve CD4^+^ T cells were differentiated for 12 days in the presence of supernatants of TLR2/1-stimulated 1,25D^diff^-DCs or serum-DCs, or without addition of DC supernatants (beads only) as described in Fig 4. After five days, rIL2 was added to all cultures. On day 12, T cells were re-stimulated using PMA/Ionomycin in fresh media the last 2.5 hours in the presence of Brefeldin A for evaluation of intracellular cytokine expression. For cytokine secretion after 18–24 hours no Brefeldin A was added. **(A)** Levels of T cell-derived IL-4 assessed by ELISA (mean of cytokine levels in ng/ml ± SEM, n = 13). **(B)** Frequency of total IL-4^+^ T cells (mean percentage of positive cells ± SEM, n = 5) and **(C)** frequency of IL-22^+^/IL-4^+^ T cells assessed by intracellular cytokine staining (mean percentage of positive cells ± SEM, n = 5). **(D)** Level of T cell-derived IL-10 assessed by ELISA (mean of cytokine levels in ng/ml ± SEM, n = 13). **(E)** Level of T cell-derived TNF-α assessed by CBA (mean of cytokine levels in ng/ml ± SEM, n = 3). *p<0.05
(EPS)

S6 Fig Expression of IL-22, IL-17a and IFN-γ in freshly isolated CD4^+^ naïve T cells. Freshly isolated naïve CD4^+^ T cells were directly stimulated with PMA/Ionomycin or left untreated in media containing 10% human AB serum for five hours, the last 2.5 hours in the presence of Brefeldin A (as described in Fig 4). T cells were stained for intracellular IL-22/IL-17a- or IL-22/IFN-γ-expression. **(A)** Frequency of total IL-22-, IL-17a- and IFN-γ-expressing CD4^+^ T cells assessed by intracellular cytokine staining (mean percentage of positive cells ± SEM, n = 5). **(B)** Dot plots from one representative staining of one donor out of five. Upper panel of dot plots shows co-expression of IL-17a and IL-22, lower panel shows co-expression of IFN-γ and IL-22. Numbers above each dot plot indicate frequency of positive cells in each quadrant. **p<0.01, ***p<0.001
(EPS)

S7 Fig Absolute T cell numbers after CD3/CD28-mediated T cell differentiation protocols. Freshly isolated naïve CD4^+^ T cells were activated with CD3/CD28-coated beads in the presence of TLR2/1-induced serum-DC (white bar) or 1,25D^diff^-DCs supernatant (black bar) or absence of supernatant (beads only control, grey bar). After five days, rIL2 was added to all cultures. On day 12, T cells were re-stimulated with PMA/Ionomycin in fresh media and counted with Trypan blue exclusion prior to intracellular cytokine staining. The asterisks directly above the bars indicate the p-values calculated in comparison to day 0 (mean of absolute cell counts per well ± SEM, n = 7). *p<0.05 and **p<0.01
(EPS)
